# A Rapid Method for the Extraction and Analysis of Carotenoids and Other Hydrophobic Substances Suitable for Systems Biology Studies with Photosynthetic Bacteria

**DOI:** 10.3390/metabo3040912

**Published:** 2013-10-11

**Authors:** Judit Bóna-Lovász, Aron Bóna, Michael Ederer, Oliver Sawodny, Robin Ghosh

**Affiliations:** 1 Institute for System Dynamics, University of Stuttgart, Pfaffenwaldring 9, Stuttgart D-70569, Germany; E-Mails: judit.bona-lovasz@isys.uni-stuttgart.de (J.B.-L.); michael.ederer@isys.uni-stuttgart.de (M.E.); oliver.sawodny@isys.uni-stuttgart.de (O.S.); 2 Institute of Theoretical Chemistry, University of Stuttgart, Pfaffenwaldring 55, Stuttgart D-70569, Germany; E-Mail: bona@theochem.uni-stuttgart.de; 3 Department of Bioenergetics, Institute of Biology, University of Stuttgart, Pfaffenwaldring 57, Stuttgart D-70569, Germany

**Keywords:** bacteriochlorophyll, carotenoids, HPLC-MS, *Rhodospirillum rubrum*, systems biology, photosynthesis, quinones, phospholipids

## Abstract

A simple, rapid, and inexpensive extraction method for carotenoids and other non-polar compounds present in phototrophic bacteria has been developed. The method, which has been extensively tested on the phototrophic purple non-sulphur bacterium *Rhodospirillum rubrum*, is suitable for extracting large numbers of samples, which is common in systems biology studies, and yields material suitable for subsequent analysis using HPLC and mass spectroscopy. The procedure is particularly suitable for carotenoids and other terpenoids, including quinones, bacteriochlorophyll *a* and bacteriopheophytin *a*, and is also useful for the analysis of polar phospholipids. The extraction procedure requires only a single step extraction with a hexane/methanol/water mixture, followed by HPLC using a Spherisorb C18 column, with a mobile phase consisting of acetone-water and a non-linear gradient of 50%–100% acetone. The method was employed for examining the carotenoid composition observed during microaerophilic growth of *R. rubrum* strains, and was able to determine 18 carotenoids, 4 isoprenoid-quinones, bacteriochlorophyll *a* and bacteriopheophytin *a* as well as four different phosphatidylglycerol species of different acyl chain compositions. The analytical procedure was used to examine the dynamics of carotenoid biosynthesis in the major and minor pathways operating simultaneously in a carotenoid biosynthesis mutant of *R. rubrum*.

## 1. Introduction

Rapid methods for chemical analysis of metabolic intermediates are becoming increasingly important in many areas, in particular for systems biology studies of the cellular metabolome. An extremely active area here is the optimization of terpenoid metabolism in bacteria, where pathway design principles are employed to achieve overproduction of industrially interesting carotenoids and quinones. In general, this area is confounded by the necessity to perform organic extractions of cellular material, for which methods are well established in the literature. However, in recent years, the widespread use of HPLC methods directly coupled to mass spectrometric (MS) analysis has presented challenging requirements for the selection of extraction solvents which are compatible with both HPLC and MS methods.

Recently, general methods for the extraction of carotenoids and quinones which are also compatible with HPLC-MS analysis have been presented [1,2]. These methods have been shown to be highly efficient and can be used for the extraction of terpenoids from a wide variety of cellular sources. Unfortunately, these latter methods have significant disadvantages for some applications. In particular, they require many extraction steps, including a cell breakage step, which becomes prohibitive for the large number of samples which are often required in systems biology studies of metabolism. For this reason we introduce here a very simple one-step procedure which is capable of extracting terpenoids as well as most other hydrophobic cellular substances present in bacteria efficiently, using a ternary solvent system (hexane/methanol/water) compatible with modern HPLC-MS analytical procedures. This method is closely related to the hexane-isopropanol [3,4,5] and hexane-ethanol [6,7] solvent systems, which are effective extraction methods without halogen-containing solutes, but is more efficient for the extraction of the special combination of hydrophobic compounds (in particular, pigments) present in photosynthetic bacteria.

Our principle motivation for introducing a new carotenoid extraction and analysis procedure is our interest in monitoring the dynamics of terpenoid metabolism for subjection to system biology analysis. We have recently demonstrated for the first time that the phototrophic bacterium, *Rhodospirillum rubrum*, which normally produces the purple carotenoid spirilloxanthin (spx) almost exclusively, can be genetically modified to overproduce the carotenoid lycopene, a carotenoid that normally only occurs at very low amounts in this organism [8]. This proof-of-principle study opens up a new genetic strategy for the production of carotenoids of industrial interest. However, rational design of new carotenoid pathways in phototrophic bacteria is complicated by the combinatorial activities of the carotenoid biosynthesis enzymes, and necessitates more detailed studies of carotenoid biosynthesis dynamics. In this context, we demonstrate the power of the extraction and analytical procedure introduced here by showing a first analysis of carotenoid biosynthesis dynamics in a mutant of *R. rubrum*, which contains a Tn*5* lesion in the *crtD* gene (rhodopin-3,4-desaturase), and produces a number of non-natural brown carotenoids via competing combinatorial pathways [9].

## 2. Results and Discussion

### 2.1. Development of a Rapid Extraction Procedure Compatible with the HPLC-APCI Analysis

Since the final goal was to develop a method for the manual extraction of hydrophobic compounds from large numbers of samples, we avoided halogen-containing solvents which, though efficient, are usually toxic and not environmentally friendly. Our initial extraction trials with the commonly used solvents, such as acetone, methanol or hexane alone, proved unsatisfactory. Acetone-methanol and hexane-ethanol mixtures showed low efficiencies of extraction for many hydrophobic but non-terpenoid molecules of interest (e.g., chlorins). Hexane, which is a good solvent for terpenoids, was inefficient for the co-extraction of phospholipids, as well as bacteriochlorophyll *a* (BChl*a*) and bacteriopheophytin *a* (BPh*a*). After various trials, we discovered that the ternary solvent methanol (MeOH)/hexane/H_2_O was extremely efficient for one-step extraction of carotenoids and quinones as well as other hydrophobic substances of interest in phototrophic bacteria, e.g., BChl*a*, BPh*a* as well as the major phospholipids.

### 2.2. Optimization of the HPLC Gradient and APCI-MS Parameters

Many HPLC separations of carotenoids have been reported previously, using a wide variety of mobile phases, typically consisting of complex binary or ternary solvent mixtures of methyl *tert*-butyl ether, acetone, acetonitrile, methanol, ethyl acetate, hexane, and tetrahydrofuran [10,11,12,13,14,15,16,17]. In general, due to the low ionization of carotenoids, atmospheric pressure chemical ionization (APCI) has become the most widely used ionization technique for MS signal detection with high sensitivity. There are also several methods for chromatographic separation and for mass spectrometric detection of phospholipids (PLs). The common ion source is electrospray ionization (ESI), but also APCI is available. These ion sources combined with MS/MS provide structural information for the intact molecule as well as the possibility of obtaining molecule-related fragment ions by in-source fragmentation or by collision-induced dissociation (CID) [18,19,20]. Many analytical methods have been reported for the analysis of isoprenoid quinones. These compounds are composed of a hydrophilic head group and an apolar isoprenoid side chain, giving the molecules a lipid-soluble character. The detection of isoprenoid quinones is usually based on HPLC analysis with UV, electrochemical or MS detection, of which MS is the most superb regarding its sensitivity and selectivity [21,22,23,24,25]. In addition, many HPLC-MS methods have been developed for the analysis of BChl*a* derivatives and their ion fragments [26,27].

Several mobile phases were tested and proved to be inefficient for carotenoid determination. Hexane and hexane-tetrahydrofuran (THF) mixtures gave very poor ionization and poor HPLC resolution. Acetonitrile and methyl *tert*-butyl ether were excluded because of environmental considerations. Methanol was found to be too polar for the separation of carotenoids.

**Figure 1:**
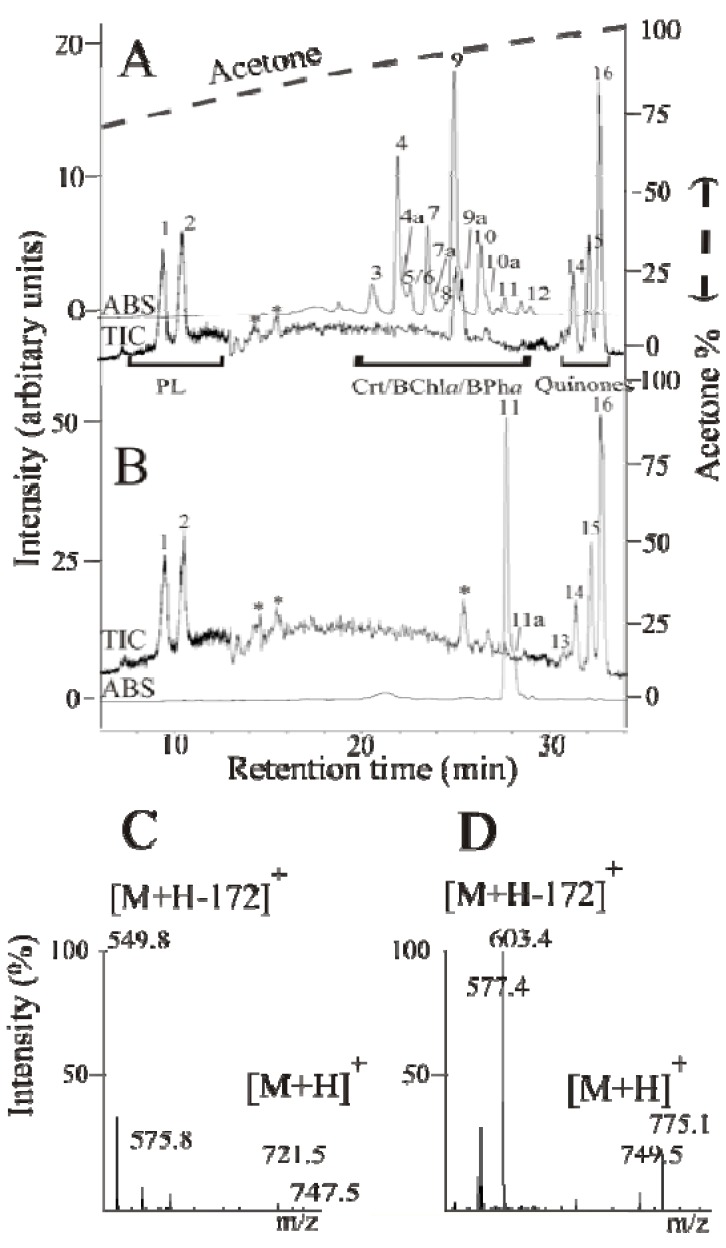
Typical HPLC-MS chromatograms of *R. rubrum* mutant strains: (**A**) the *crtD*^−^ strain ST4; and (**B**) the (*crtCD*)^−^ strain SLYC18 (see Figure 2 for the gene designations). The chromatogram shows three separation regions, corresponding to phospholipids (PLs), photosynthetic pigments (BChl*a*, BPh*a*, crt), and quinones, respectively. Peak identifications (1) PPoPG and PoOPG; (2) POPG and DOPG; (3) 1-methoxy-1′-OH-3,4-didehydrolycopene; (4) *trans*-3,4-dihydrorhodovibrin; (4a) *cis*-3,4-dihydrorhodovibrin; (5) 1-OH-3,4-didehydrolycopene; (6) 1′-OH-3,4-dihydrospheroidene; (7) *trans*-rhodopin; (7a) *cis*-rhodopin; (8) 1′-OH-neurosporene (chloroxanthin); (9) *trans*-tetrahydrospirilloxanthin (thspx); (9a) *cis*-thspx; (10) *trans*-3,4-dihydroanhydrorhodvibrin; (10a) *cis*-3,4-dihydroanhydrorhodovibrin; (11) *trans*-lycopene; (11a) *cis*-lycopene; (12) neurosporene; (13) RQ-9; (14) UQ-9; (15) RQ-10; (16) UQ-10. The absorption at 475 nm (ABS) and total ion counts (TIC) channels between m/z 350–1,000 are indicated. The CID fragmentation patterns of PL peaks (1) and (2) at 75 fragmentor voltage are shown in (**C**) and (**D**), respectively. Base peaks are the DAG_MS_ fragments (549.4/575.4). A fatty acid fragment with its glycerol backbone is also observable in the extended MS profile with lower intensities. [M+74]^+^: 339 = oleic acid, 311 = palmiteolic acid (data not shown). Peaks marked by asterisks are due to three minor contaminants present in the elution solvent. For the SLYC18 profile, the third minor contaminant coelutes with lycopene.

We discovered that a non-linear acetone gradient was eminently suitable for separating a wide variety of non-polar compounds, including photosynthetic pigments, as well as being suited to subsequent APCI analysis. Typical HPLC runs performed with extracted cells from two carotenoid biosynthesis mutants, ST4 (*crtD*^−^) and SLYC18 ((*crtCD*)^−^), respectively, are shown in Figure 1A,B. As indicated in the Figure 1, three major separation regions, corresponding to phospholipids, photosynthetic pigments (carotenoids (crt), BChl*a* as well as BPh*a*), and quinones, respectively, are clearly distinguishable and the gradient is able to achieve the necessary separation of all compounds required for their subsequent identification by on-line APCI-MS analysis. A full analysis of the results obtained from the *R. rubrum* strains is presented below.

In the experiments described in this study, we achieved the separation of 18 carotenoids with one elution profile. In the carotenoid region, the gradient was adjusted to be flatter, since carotenoids have a wide range of components and are difficult to separate. Coelution occurred only between ubiquinol-10 (UQH_2_-10) and ubiquinone-9 (UQ-9) as well as between two of the phosphatidylglycerols (PGs).

The ionization parameters were optimized for carotenoids, since they are the most non-ionizable molecules. The parameters, temperature and fragmentor voltage, provide the greatest influence on the ionization for each class of compound shown in Figure 1A,B. Carotenoids and isoprenoid-quinones are not fragmented at the fragmentor voltage of 75 V, but form a molecular ion by accepting a proton. All major terpenoid quinones, such as rhodoquinone-9 (RQ-9), rhodoquinone-10 (RQ-10), UQ-9, UQ-10 and UQH_2_-10 showed retention times of 31.6–33.5 min. Although UQH_2_-10 co-elutes with RQ-9, the molecular ions are distinguishable.

The detection of BChl*a* was supported by UV-VIS detection at 772 nm. The molecular ion [M+H]^+^ of BChl*a* (905.4) occurs with very low intensity in the sample. We also found the fragment ion *m/z* = 889.4, which corresponds to the mass of protonated BPh*a*. Geranylgeranyl-BPh*a* was also detected at low intensity with the base beak 883.5. In control experiments using purified BChl*a*, we showed that a significant amount of the geranylgeranyl-derivative of BChl*a* loses the Mg^2+^ during ionization, thereby generating the geranylgeranyl derivative of BPh*a*. For *R. rubrum*, it has been shown that BChl*a* is exclusively present as the geranylgeranylderivative [28] whereas naturally occurring BPh*a*, which is tightly bound to the photosynthetic reaction center, is present as the phytyl derivative [29].

The two peaks appearing at about 10 min in the HPLC profile (Figure 1A,B) each comprised of two phospholipids, which were identified unambiguously in the mass profile as PGs. Peak 1 contained palmitoleyl (C16:1)-oleoyl (C18:1)-phosphatidylglycerol ((PoOPG, *m/z* = 747.5) and palmitoyl (C16:0)-palmitoleyl-PG (PPoPG, *m/z* = 721.5), where PPoPG was the predominating phospholipid. The fragmentation of PGs in positive mode led to a [M+H-172]^+^ ion obtained from the neutral loss of the PG head group, resulting in a detection of diglycerides (DAGs) as diglycerol-like (DAG_MS_) fragments [18]. The fatty acid residues were thus identified as their DAG_MS_ fragments (*m/z* (C18:1/C16:1) = 575.4; *m/z* (C16:1/C16:0) = 549.4). Peak 2 contained dioleoyl-PG (DOPG, *m/z* = 775.1) and palmitoyl-oleoyl-PG (POPG, *m/z* = 749.5), which also yielded the corresponding DAG_MS_ fragments (*m/z* (C18:1/C18:1) = 603.4; *m/z* (C16:0/C18:1) = 577.4), respectively, in the MS profile. Phosphatidylethanolamine, which is the major phospholipid in *R. rubrum*, comprising about 80% of the total phospholipid [30,31], can be detected as a broad peak of low intensity, eluting between 10–25 min. By contrast, PG comprises about 17% of the total phospholipid under both phototrophic as well as dark, semi-aerobic conditions [30,31]. The fatty acid analysis of the PGs in Figure 1C,D is consistent with those observed using classical extraction procedures in the literature [31,32,33,34,35,36]. In all of the aforementioned studies, the predominant fatty acids in *R. rubrum* under all growth conditions are C16:0, C16:1, and C18:1, with the latter comprising about 10% more than either C16:0 or C16:1 (although the absolute values vary somewhat depending upon the growth conditions as well as the extraction procedure used). If we assume that all the PG fatty acids are observable in the MS profiles, a preliminary calculation indicates that the PG produced under the dark, semi-aerobic conditions employed here, is principally constituted from C16:0, C16:1, and C18:1 fatty acids in the ratio 32.8%, 25.0%, and 42.4% by weight, respectively. These ratios are very similar to those observed by Russell and Harwood [36] for *R. rubrum* growing under dark, semi-aerobic conditions.

### 2.3. Detailed Analysis of the Carotenoids Present in the Mutant Strains ST4 and SLYC18

**Figure 2:**
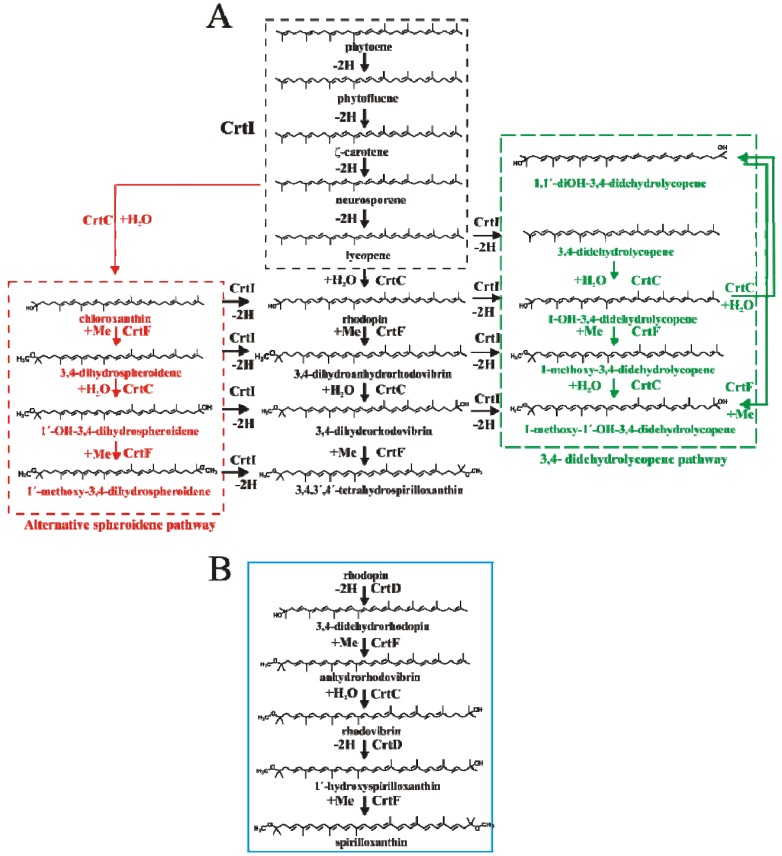
(**A**) The carotenoid biosynthetic pathway in the *crtD*^−^ mutant *R. rubrum* ST4. The central pathway (shown in black) represents the linear sequence of metabolic reactions as established for the wild-type pathway to spirilloxanthin [37,38], but lacking the 3,4- and 3′,4′-dehydrogenation steps catalyzed by CrtD [9]. The minor pathways, the alternative spheroidene pathway and the 3,4-didehydrolycopene pathway, are shown in the red and green boxes, respectively. The putative CrtI branch points linking the major and minor pathways are indicated. (**B**) The wild-type carotenoid biosynthetic pathway following the intermediate, rhodopin, is shown in the blue box.

We illustrate the power of the HPLC method here using the carotenoid mutants ST4 and SLYC18 [8]. It is well-established that the carotenoid biosynthesis pathway in the wild-type strain S1 (see Figure 2) shows a linear reaction sequence, whereby lycopene is produced via 4 sequential dehydrogenation steps from phytoene by a single enzyme, CrtI, and thereafter, hydration (catalyzed by CrtC), 3,4-/3′-4′-dehydrogenation (catalyzed by CrtD) and methylation (catalyzed by CrtF) are performed sequentially on both ends of the carotenoid molecule, to yield the final product, spirilloxanthin [37,38,39]. Carotenoid analysis of S1 at different stages of growth using our HPLC method were consistent with a linear reaction sequence leading to spx, with intermediate carotenoids occurring significantly only in rapidly growing cells (data not shown). This result has also been observed previously [39]. However, as first demonstrated by Komori *et al.* [9], deletion of *crtD* leads to the appearance of alternative side pathways, due to the lack of absolute substrate specificity of the carotenoid biosynthesis enzymes. Thus, as indicated in Figure 2A, in the absence of CrtD (which normally dehydrogenates rhodopin, and rhodovibrin, leading to 3,4-didehydrorhodopin and monomethylated spirilloxanthin, respectively), the enzyme CrtF is able to methylate rhodopin, yielding the non-natural carotenoid 3,4-dihydroanhydrorhodovibrin. The latter can also act as a non-natural substrate for CrtC which yields 3,4-dihydrorhodovibrin, which in turn acts as a non-natural substrate for CrtF, to yield the final product, thspx (Figure 2A, pathway in black). For ST4 cells grown either phototrophically or semi-aerobically, thspx is the predominant (about 80% of the total) carotenoid produced in the photosynthetic membrane in the late exponential growth phase [9]. The observation of the non-natural thspx pathway, which arises solely due to the deletion of *crtD*, confirmed unambiguously that the substrate specificity of the carotenoid biosynthesis enzymes is broad, and that they can act combinatorially upon available substrates. In recent years, the broad substrate specificity, which allows “sequential” reactions to occur combinatorially (provided that the chemistry of the reactions remains unchanged), has been used for the biotechnological production of non-natural carotenoids [40,41].

Figure 1A shows a typical HPLC profile of extracted carotenoids present in ST4 cells growing exponentially under dark, semi-aerobic conditions. In order to yield the maximum number of carotenoids arising from major and side pathways, we found it necessary to grow the cells in a special regime of oxygen-limitation (see Materials and Methods for details). Nevertheless, consistent with the results of Komori *et al.* [9], who extracted carotenoids from phototrophically-grown stationary-state cells, thspx is the major carotenoid at the end of the growth phase.

Under the growth conditions used here, we can detect clear evidence for two alternative pathways in addition to the major linear reaction sequence produced by the sequential action of the enzymes CrtC and CrtF upon lycopene (shown as the central block in Figure 2A).

The observation of chloroxanthin (1-OH-neurosporene, Figure 1A, peak 8), can only appear due to the action of CrtC on neurosporene (one of the CrtI intermediates). Chloroxanthin can clearly act as a non-natural substrate for CrtF, as evidenced by the appearance of 1′-OH-3,4-dihydrospheroidene (Figure 1, peak 6, Table 1). The latter product arises from the action of CrtC upon 3,4-dihydrospheroidene. However, 3,4-dihydrospheroidene was not observed, presumably because the CrtC-mediated reaction is much more efficient than that of CrtF under O_2_-limiting conditions (see below). A further possible intermediate, 1′-methoxy-3,4-dihydrospheroidene, which could arise by the action of CrtF upon the last product, was not observed in this experiment. The appearance of this side pathway, designated here the alternative spheroidene pathway (Figure 2A, pathway in red), implies that most or all of the intermediates are still possible substrates for CrtI, since thspx is still produced as the major carotenoid in the late phase of growth (see below for a description of the dynamics of the system).

**Table 1:** List of identified compounds in *R. rubrum*.

	Compound	Retention time (min)	Exact Mass (g)	Molecular ion	Molec. ion/fragment ^a^
**Phospholipids**	Palmitoyl-palmitoleyl-PG (C16:0/C16:1) (PPoPG)	9.14	720.5	[M+H-172]^+^	549.4
	Palmitoleyl-oleoyl-PG (C16:1/C18:1) (PoOPG)	9.14	746.5	[M+H-172]^+^	575.4
	Palmitoyl-oleoyl-PG (C16:0/C18:1) (POPG)	9.95	748.5	[M+H-172]^+^	577.4
	*Pentadecanoyl-octadecenoyl-PG* (C15:0/C18:1)	9.95	734.5	[M+H-172]^+^	563.4
	*Tetradecanoyl-hexadecanoyl-PG* (C14:0/C16:0)	9.95	694.5	[M+H-172]^+^	523.4
	*Heptadecenoyl-octadecenoyl-PG* (C17:1/C18:1)	9.95	760.5	[M+H-172]^+^	589.4
	*Octadecenoyl-nonadecenoyl-PG* (C18:1/C19:1)	9.95	788.5	[M+H-172]^+^	617.4
	*Tridecanoyl-octadecenoyl -PG* (C13:0/C18:1)	9.95	706.5	[M+H-172]^+^	535.4
	*Dioctadecanoyl-PG* (C18:0/C18:0)	9.95	778.5	[M+H-172]^+^	607.4
	*Octadecanoyl-nonadecanoyl-PG* (C18:0/C19:0)	9.95	792.5	[M+H-172]^+^	621.4
	Dioleoyl-PG (C18:1/C18:1) (DOPG)	9.95	774.5	[M+H-172]^+^	603.4
**Carotenoids/BChla/BPha**	BChl*a* (geranylgeranyl)	19.06	904.5	[M+H]^+^	905.5
	Rhodovibrin	21.43	584.5	[M+H]^+^	585.5
	1,1′-Dihydroxy-3,4-didehydrolycopene	21.43	570.5	[M+H]^+^	571.5
	1′-Hydroxyspirilloxanthin	20.85	582.5	[M+H]^+^	583.5
	1-Methoxy-1′-OH-3,4-didehydrolycopene	20.67	584.5	[M+H]^+^	585.5
	3,4-Dihydrorhodovibrin	22.07	586.5	[M+H]^+^	587.5
	1-Hydroxy-3,4-didehydrolycopene	22.24	552.5	[M+H]^+^	553.5
	3,4-Didehydrorhodopin	22.07	552.5	[M+H]^+^	553.4
	Chloroxanthin (1-OH-neurosporene))	24.61	556.5	[M+H]^+^	557.5
	*BPha (geranylgeranyl)*	23.60	882.5	[M+H]^+^	883.5
	Rhodopin	23.63	554.4	[M+H]^+^	555.4
	Spirilloxanthin	23.87	596.4	[M+H]^+^	597.4
	3,4-Dihydrospirilloxanthin	24.46	598.5	[M+H]^+^	599.5
	3,4,3′,4′-Tetrahydrospirilloxanthin	25.05	601.4	[M+H]^+^	602.4
	BPh*a* (phytyl)	26.75	888.5	[M+H]^+^	889.5
	1′-Hydroxy-3,4-dihydrospheroidene	22.72	588.5	[M+H]^+^	589.5
	Anhydrorhodovibrin	25.91	566.5	[M+H]^+^	567.5
	3,4-Dihydroanhydrorhodovibrin	26.74	568.5	[M+H]^+^	569.5
	Phytoene	31.25	544.5	[M+H]^+^	545.5
	Lycopene	27.75	536.5	[M+H]^+^	537.5
	*Neurosporene*	29.11	538.5	[M+H]^+^	539.5
	Rhodoquinone-9 (RQ-9)	30.77	779.6	[M+H]^+^	780.6
**Quinones**	Ubiquinol-10 (UQH_2_-10)	31.08	864.7	[M+H]^+^	865.7
	Ubiquinone-9 (UQ-9)	31.43	794.6	[M+H]^+^	795.6
	Rhodoquinone-10 (RQ-10)	32.24	847.7	[M+H]^+^	848.7
	Ubiquinone-10 (UQ-10)	32.81	862.7	[M+H]^+^	863.7

^a^ The molecular ion fragments arising from the PGs corresponds to the calculated mass of the diglyceride-like (DAG_MS_) species [18]. Species shown in italics were only detected in trace amounts. For the predominant PGs, we have retained the common names to aid readability for a broader audience, whereas for the trace PGs we have used the systematic names.

The observation of the carotenoids 1-OH-3,4-didehydrolycopene (Figure 1, peak 5) and 1-methoxy-1′-OH-didehydrolycopene (Figure 1, peak 3) indicates that a second minor pathway (here designated the 3,4-didehydrolycopene pathway (Figure 2, pathway in green)) is also operative under oxygen-limiting conditions. 1-methoxy-1′-OH-didehydrolycopene can arise via three potential routes, two of which occur via a CrtI-mediated side reaction and one of which is due to the action of CrtF and CrtC on the CrtI-mediated intermediate 1-OH-3,4-didehydrolycopene (Figure 2). The CrtI-mediated reactions are probably minor (as the main thspx pathway is predominant), so we propose that the concerted action of CrtF and CrtC is the principle pathway. However, the obligatory intermediate of this latter pathway, 1-methoxy-3,4-didehydrolycopene, was not detected, presumably because the CrtC-mediated reaction is faster than the CrtF reaction under oxygen-limited conditions (see below).

In contrast, Komori *et al.* [9] detected only 1,1′-diOH-lycopene as a minor component in the carotenoid extracts from anaerobic, phototrophically-grown ST4 cells. However, in our experiments (using semi-aerobically-grown ST4 cells) this latter intermediate was not observed. We hypothesize that this difference is due to the different growth conditions employed in the study of [9] and those reported here.

We deduce that the 3,4-dehydrogenation step, in the absence of CrtD, which normally catalyzes this reaction *in vivo*, is due to the action of CrtI, since this is the only carotenoid desaturase present in the strain ST4. In fact, several groups [39,41] have shown that fungal CrtIs overexpressed in *E. coli* can catalyze a 3,4- as well as 3′,4′-desaturation step, though very inefficiently. Although the desaturation of a lycopene derivative containing a 1- or 1′-tetrahedral substituent (e.g., rhodopin, which possesses a 1-hydroxy-substituent) has to our knowledge not been reported so far, the observation of 1-OH-3,4-didehydrolycopene is evidence that the *R. rubrum* CrtI can perform this reaction, albeit at very low levels. The absence of 3,4-didehydrolycopene in both ST4 and the lycopene-producing mutant SLYC18, suggests that, under our growth conditions, the direct dehydrogenation of lycopene by CrtI does not occur. Nevertheless, it seems also likely that the methoxy-derivatives of rhodopin can also be dehydrogenated by CrtI. We therefore have indicated putative CrtI-mediated branch points connecting the major pathway to the minor 3,4-didehydrolycopene pathway in Figure 2.

The HPLC profile of cell extracts of the lycopene-producing mutant SLYC18 (Figure 1B) provides an interesting control experiment. Since this mutant lacks both CrtC and CrtD, none of the carotenoids beyond lycopene can be present. The HPLC profile indeed shows lycopene as the only major carotenoid present (Figure 1B, peak 11), although very low amounts of neurosporene are also observable (very minor peak situated to the right of peak 11 in Figure 1B). We have shown recently that lycopene in SLYC18 is sequestered exclusively in the light-harvesting 1 complexes immediately following its production by CrtI and does not accumulate on the carotenoid biosynthetic complex [8]. Thus, the presence of the alternative spheroidene pathway in ST4 is presumably due to the accumulation of lycopene on CrtI during oxygen-limiting conditions, which is due to product inhibition of the enzyme, and causes a small amount of neurosporene to be released for further biosynthetic processing.

The HPLC profile of SLYC18 also provides a useful control of the extraction procedure. Thus, the amounts of the observable phospholipids and quinones, respectively, appear to be essentially identical in the profiles of SLYC18 and ST4, confirming that these substances are extracted reproducibly.

### 2.4. HPLC-MS Analysis of Carotenoid Pathway Dynamics

**Figure 3:**
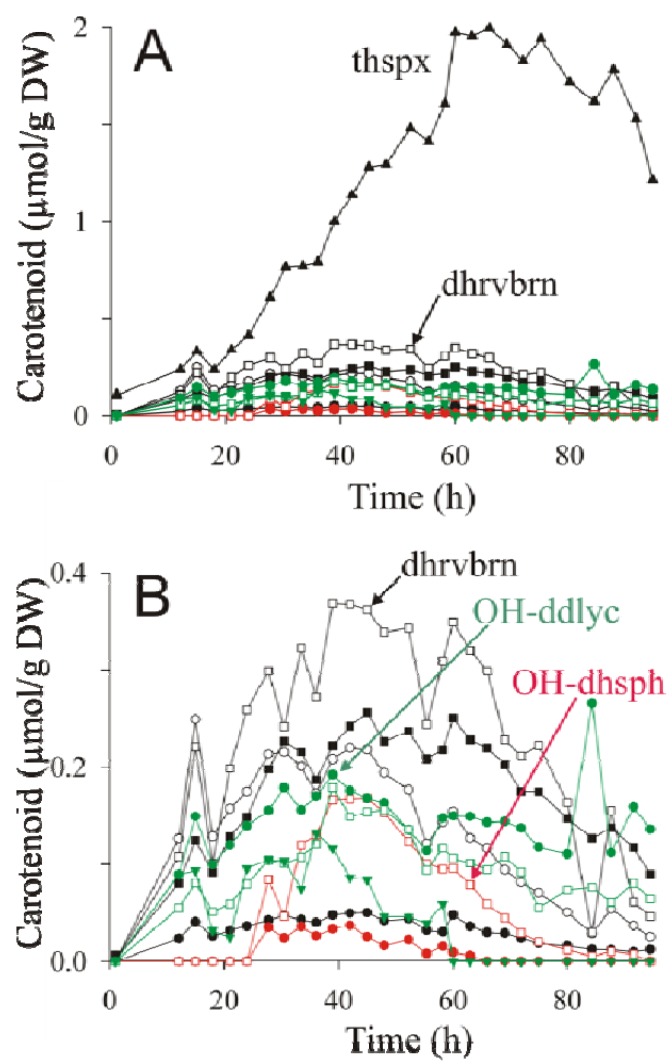
Primary data for dynamic changes of various carotenoid species. The figure shows LC-MS data from extracts of ST4 obtained at various time points of a semi-aerobic growth curve performed under an argon atmosphere. The data for all of the carotenoids determined are shown in (**A**). The same data, excluding thspx, have been plotted with an expanded scale (**B**) for clarity. Abbreviations: thspx (▲); 3,4-dihydrorhodovibrin (dhrvbn) (□); rhodopin (○); 3,4-dihydroanhydrorhodovibrin (■); lycopene (●); chloroxanthin (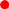); 1′-OH-3,4-dihydrospheroidene (OH-dhsph) (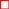); 1 -OH-3,4-didehydrolycopene (OH-ddlyc) (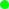); 1-methoxy-1′-OH-didehydrolycopene (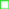); 1,1′-diOH-3,4- didehydrolycopene (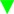).

The large variety of carotenoids produced by ST4 under semi-aerobic conditions provides a useful example to illustrate the power of the present rapid isolation and analysis method for examining carotenoid pathway dynamics. The strain ST4 was therefore grown semi-aerobically as described above, and carotenoid extractions were performed throughout the growth phase, and then quantified by HPLC-MS analysis. The results of the raw data are shown in Figure 3A,B.

As previously noted by Komori *et al.* [9], thspx dominates the carotenoid composition towards the end of the growth curve (Figure 3A). However, during the early phase of exponential growth, the intermediates of the thspx pathway, as well as those of the minor alternative pathways contribute significantly to the total carotenoid composition. We note that the abrupt breaks of the carotenoid profiles are due partly to the technical difficulties of sampling a liquid shake culture under argon. In our manual sampling procedure, it is very difficult to avoid the introduction of a small pulse of oxygen during sampling, which probably causes a rapid, though transient, inactivation of carotenoid biosynthesis subsequent to the sampling event.

The dynamics of the carotenoid synthesis pathways can be visualized more effectively by calculating the “fraction carotenoid” from the raw data as:

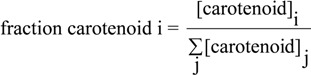
(1)
where [carotenoid]_i_ is the concentration of a given carotenoid species i at time point t, and the sum 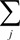 is the total concentration of all carotenoid species found at time point t.

**Figure 4:**
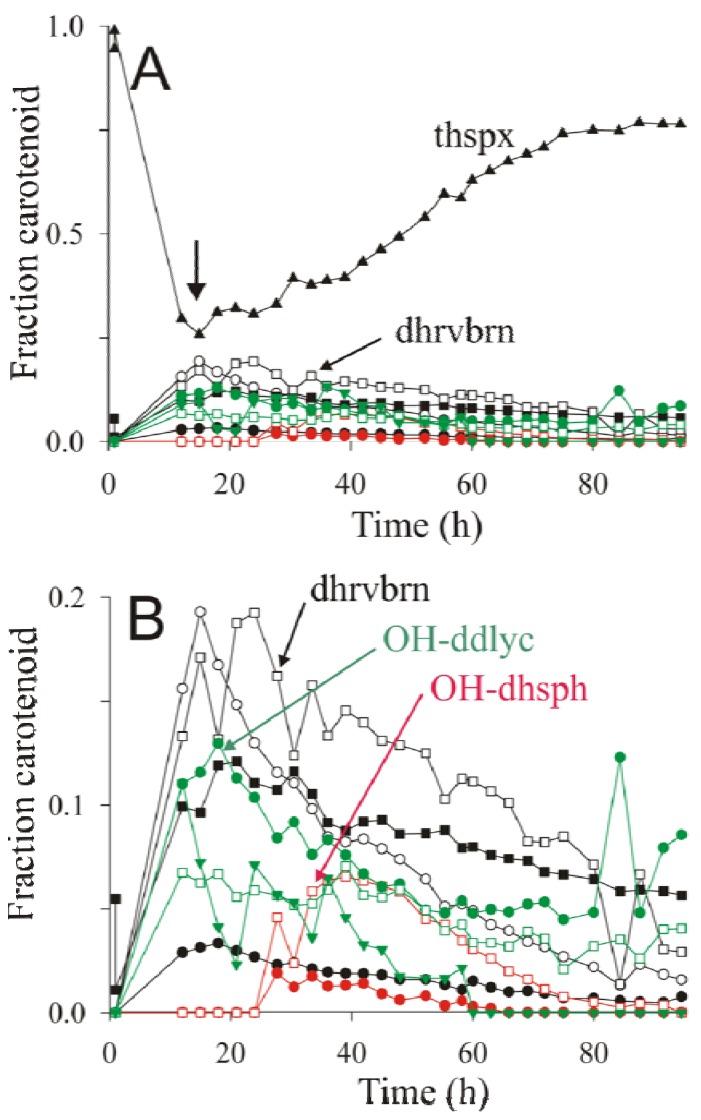
The fractional profiles of carotenoid dynamics calculated from the primary dynamic data shown in Figure 3.

The re-calculation of the raw data according to Equation (1) is shown in Figure 4A,B.

The fractional profiles were calculated using Equation (1). The symbol code is the same as in Figure 3.

For the purpose of visualizing the dynamics of carotenoid biosynthesis, the calculation of the fraction carotenoid has two advantages over the direct visualization of the primary data. First, systematic sampling errors are largely “cancelled out”, thereby causing a smoothing of the data. Secondly, whereas the primary data value “μmol carotenoid/g DW” is essential for calculating the yield of carotenoid produced, it also contains contributions from extraneous factors (e.g., systematic sampling errors, changes of cell morphology or composition during growth), which make intuitive evaluation of the dynamics difficult. For instance, in Figure 3A, the thspx level seems to decrease at the end of the growth curve, whereas in Figure 4A the thspx level rises continously to an asymptotic value. The reason for this discrepancy is probably due to the fact that *R. rubrum* cells show significant lengthening in the late exponential phase, probably associated with substrate limitation, with a non-proportional increase in the biomass [8], thus causing the amount of carotenoid/g DW cell to decrease. However, the data in Figure 4A shows that the relative amount of carotenoid with respect to the total remains unchanged within the lengthening phase. We note though, that carotenoid metabolism is also halted in this late growth phase.

Analysis of the data in Figure 4A,B allows the temporal behavior of the major (thspx) and minor pathways to be assessed visually. For instance, at early times, the thspx level (initially due to the inoculum) drops continuously until about 18 h, after which it rises continuously. It is now well-established that the apparent decrease in photosynthetic pigments in the early growth phase is due to repression of their biosynthesis genes by oxygen [42] and growth-induced dilution of photosynthetic membranes/cell. For *R. rubrum*, when a critical pO_2_ of <0.3% is attained (due to the cellular oxygen consumption under oxygen-limited semi-aerobic conditions), the oxygen-mediated repression is abolished, allowing the biosynthesis of photosynthetic pigments. This leads to the “trough” (indicated by an arrow in Figure 4A) in the profiles of the photosynthetic pigments such as BChl*a* [43] and carotenoids (this study). Comparison of the position of the trough for thspx (at 18 h) and the appearance of the carotenoid intermediates shows that the highest intermediate of the main thspx pathway at 18 h corresponds to rhodopin (the third intermediate prior to thspx), followed closely by 3,4-dihydrorhodovibrin (the first intermediate prior to thspx), whereas the second intermediate prior to thspx, 3,4-dihydroanhydrorhodovibrin, is somewhat lower in fractional concentration at this time point. This apparently contradictory appearance of intermediates in a linear pathway, indicates that the CrtF-mediated reactions are limiting compared to the CrtC-mediated reactions, thus causing a build-up (at early times) of the appropriate intermediates. This hypothesis is also consistent with the low concentration of lycopene, which is rapidly converted to rhodopin by an efficient CrtC-mediated reaction (Figure 4B).

The accumulation of the 3,4-dihydroanhydrorhodovibrin and 3,4-dihydrorhodovibrin on the main pathway would explain the "spill-over" into the minor 3,4-didehydrolycopene pathway, presumably due to the action of CrtI upon these intermediates. Although the role of CrtI in this context has not been demonstrated unambiguously, in the absence of CrtD, CrtI is the only carotenoid desaturase present in the genome which might catalyze the cross-over reactions to the side pathway.

At the 18 h time point, the fractional levels of 1,1′-diOH-3,4-didehydrolycopene and 1-OH-3,4,-didehydrolycopene are closely similar, which again suggests that the CrtF-mediated reaction (here leading to 1-methoxy-3,4-didehydrolycopene) is rate limiting. However, at late times, the intermediate 1,1′-diOH-3,4-didehydrolycopene falls to undetectable levels, presumably due to CrtF-mediated methylation to form 1-methoxy-1′-OH-3,4-didehydrolycopene (Figure 2A). The latter intermediate remains at low but significant levels until the end of the growth curve, as does the first minor pathway intermediate, 1-OH-3,4-didehydrolycopene. In contrast, the CrtF-mediated intermediate, 1-methoxy-3,4-didehydrolycopene was not detected, presumably because low levels of this carotenoid are rapidly removed by the efficient CrtC-mediated reaction step (Figure 2A). In summary, these observations indicate that CrtF possesses an “end” specificity, as is also indicated by the analysis of the thspx major pathway, even though the enzyme can act combinatorially.

Interestingly, the minor alternative spheroidene pathway (Figure 2A, red) appears only after the other pathways are decaying. In this context, it is significant that neurosporene was hardly detected, despite the fact that this intermediate is an obligatory precursor for the alternative spheroidene pathway. We note also that neurosporene was only detected in very small amounts in the lycopene-producing mutant SLYC18 [8] (see also Figure 1B). However, we have shown recently that in SLYC18, lycopene is incorporated immediately into the LH1 complex [8], and thus not able to act as a product inhibitor of CrtI. Our experiments here with ST4 indicated that lycopene begins to accumulate at about 18 h, and thus may also act as product inhibitor of CrtI, thereby leading to the release of small amounts of neurosporene, which are immediately converted (via CrtC) to chloroxanthin. This low level channelling initiates the alternative spheroidene pathway, which ultimately is rechannelled into the thspx pathway via CrtI at the level of 1′ -OH-3,4-dihydrospheroidene (as indicated by its accumulation with respect to chloroxanthin) (Figure 4B). A further possible intermediate, 1′-methoxy-3,4-dihydrospheroidene was not found, which would be consistent with the higher "end specificity" of CrtF in comparison to CrtC.

## 3. Experimental Section

### 3.1. Chemicals and Reagents

LC-MS grade hexane, GC grade acetone and MS grade water were purchased from Roth (Karlsruhe, Germany). UQ-10 was obtained from Applichem (Darmstadt, Germany). BChl*a* and dioleoyl-phosphatidylcholine (DOPC) were obtained from Sigma Aldrich (St. Louis, MO, USA). Lycopene was obtained from a lycopene-producing strain of *R. rubrum* [8].

### 3.2. Cell Growth of R. rubrum Strains

*R. rubrum* strains were batch-grown in a stirred closed flask (two replicates for each strain) under semi-aerobic conditions (pO_2_ < 0.3%) at 30 °C using a modified Sistrom medium A [44]. The modified medium, designated M2SF, contains additional succinate and fructose, as well as increased buffer capacity, and allows photosynthetic levels of pigments to be produced under semi-aerobic growth conditions [45]. For cell growth experiments for the determination of carotenoid biosynthesis dynamics, semi-aerobically-grown cells of the *R. rubrum* *crtD*^−^ mutant were grown in small shake flasks under an atmosphere of argon. Since flasks were not sealed, the leakage of oxygen through the argon layer into the medium was sufficient to maintain relatively constant O_2_-limited conditions. Samples (amounts depending on the cell density) were taken from under the argon layer approximately every 4–5 h, and the cultures were flushed with argon every 12 h. In general, the duration of the growth experiment was about 100 h.

### 3.3. Extraction Method

The sampling and extraction procedure was performed as follows: 5–20 mL bacterial fermentation broth was centrifuged at 4,000 rpm (1,800 ×g) for 15 min, then the supernatant was discarded. 1 mL methanol and 2 mL hexane were then added to the pellet, and the mixture was vortexed for 2 min. Subsequent phase separation was achieved by the addition of 1 mL of water to the mixture, followed by vortexing for 1 min. and centrifugation at 4,000 rpm for 20 min. The upper supernatant phase was passed through a nylon filter (2 µm, diameter 13 mm) (Roth, Karlsruhe, Germany), then used directly for HPLC-MS analysis. Every step of the extraction and the storage of the pellets were carried out under argon and in the dark.

### 3.4. Calibration Procedures

The standards (lycopene, UQ-10, and DOPC) were dissolved in hexane and then placed under argon. All standards were stored at −84 °C. Concentrations of lycopene stock solutions were determined spectrophotometrically using the molar extinction coefficient at 502 nm in hexane (1.72 × 10^5^ M^−1^·cm^−1^ [46]). Although the integral of the selected ion monitoring (SIM) channel (*m/z =* 537.5 [M+H]^+^) varies linearly with the lycopene concentration, the optical absorption of lycopene at 502 nm gives higher sensitivity and was routinely used for calibration. Since the magnitudes of carotenoid extinction coefficients are proportional to the number of conjugated double bonds [46], rhodopin was calibrated using the extinction coefficient for lycopene. For the other carotenoids (anhydrorhodovibrin, rhodovibrin, spx, thspx) the extinction coefficients were calculated from the extinction coefficients described in [37]. Isoprenoid-quinone calibration was done by using a commercial UQ-10 standard, and showed a linear response. This was also true for the calibration for UQ-9, since it only differs in the length of its isoprenoid chain, therefore its molar ionization rate is the same [47]. BChl*a* was calibrated using methanol extracts of *R. rubrum* cells. This extraction procedure yields mainly BChl*a* as well as small amounts of BPh*a* and some phospholipids, mainly PG. The concentration of BChl*a* was determined using the molar extinction coefficient of 6 × 10^4^ M^−1^·cm^−1^ at 772 nm [48]. For the phospholipid calibration, we utilized the fact that the phospholipid ionization rate does not depend on the fatty acid composition [47]) as well as the fact that PG, PE and phosphatidylcholine appear as DAG fragments in the mass spectrum. Thus, the phospholipid was calibrated with a phosphatidylcholine standard. The precision of the HPLC-APCI/MS method was determined from the results obtained from 30 separate extractions for lycopene, BPh*a*, and UQ-10, respectively, and those from 18 separate extractions for PLs. The coefficient of variation was under 8%, 11.7%, 16.9%, and 9.7% for carotenoids, BChl*a*, PLs, and UQ-10, respectively.

### 3.5. Instrumentation and Operating Conditions

HPLC analyses were performed on a Dionex Ultimate 3000 (Dionex Corp. (ThermoFisher Scientific), Sunnyvale, CA, USA) separation module equipped with a Dionex Ultimate (Dionex Corp.) column oven, and VWD-3000 UV-VIS (Dionex Corp.) detector controlled by a UCI-1500 universal chromatography interface (Dionex Corp.). The analytical scale Spherisorb C18 reversed-phase column (VDS Optilab, 250 mm × 4.6 mm i.d., 5 µm particle size) used was operated at 25 °C. The mobile phase consisted of acetone/water (50:50 (*v/v*)) as a starting composition using a flow rate of 1 mL/min. The non-linear gradient used, programmed using the Chromeleon 6.8 software (Dionex Corp.), is shown in Figure 1. The total run time was 45 min. and the injection volume was 20 µL. UV-VIS detection was performed at 475 nm.

The HPLC system was coupled on-line to a Dionex MSQ™ single quadrupole mass spectrometer (Dionex Corp. (ThermoFisher Scientific), Sunnyvale, CA, USA) fitted with an APCI ion source. MS control was performed using Excalibur^®^ (ThermoFisher Scientific, Sunnyvale, CA, USA) software. The instrument was operating in the positive ion mode at a fragmentor voltage of 75 V. The mass spectra of the column eluates were recorded in the range of *m/z* = 250–1,000 with a dwell time of 0.3 s. Nitrogen was used as the nebulising gas at a pressure of 100 psi. The vaporizer temperature was set at 450 °C and the corona current was set at 7.5 µA. All data were acquired in centroid mode and processed using Chromeleon^®^ 6.8 (Dionex Corp. (ThermoFisher Scientific), Sunnyvale, CA, USA) software.

## 4. Conclusions

The rapid hexane-methanol-water extraction method coupled to the acetone-water HLPC elution system presented here is eminently suitable for the analysis of a wide variety of hydrophobic compounds, in particular, terpenoids, carotenoids, and chlorins, as well as polar phospholipids, present in phototrophic bacteria. We expect the method to be also successful for most gram-negative bacteria. Apart from the rapid procedure, which sets this method apart from many others, the organic solvents used for extraction and HPLC separation are only mildly toxic, and also biodegradable, and are thus well-suited for many general biochemical laboratories.

The rapid procedure has also been shown here to yield acceptable data for studying the dynamics of a range of hydrophobic substances simultaneously, which is one of the prerequisites for mathematical modeling of metabolism using modern systems biology methods. We believe, therefore, that our method will provide a new impulse for the study of the very versatile metabolism exhibited by purple phototrophic bacteria, and facilitate their use in industrial bioengineering.
